# Site-specific impacts on gene expression and behavior in fathead minnows (*Pimephales promelas*) exposed *in situ *to streams adjacent to sewage treatment plants

**DOI:** 10.1186/1471-2105-10-S11-S11

**Published:** 2009-10-08

**Authors:** Natàlia Garcia-Reyero, Ira R Adelman, Dalma Martinović, Li Liu, Nancy D Denslow

**Affiliations:** 1 Department of Physiological Sciences and Center for Environmental and Human Toxicology, University of Florida, Gainesville, FL 32611, USA; 2 Dept. of Fisheries, Wildlife, and Conservation Biology, University of Minnesota, St. Paul, MN, USA; 3 Department of Biology, University of St Thomas, St Paul, MN, USA; 4 ICBR, University of Florida, Gainesville, FL 32611, USA; 5 Department of Chemistry, Jackson State University, Jackson, MS 39217, USA

## Abstract

**Background:**

Environmental monitoring for pharmaceuticals and endocrine disruptors in the aquatic environment traditionally employs a variety of methods including analytical chemistry, as well as a variety of histological and biochemical endpoints that correlate with the fish fitness. It is now clear that analytical chemistry alone is insufficient to identify aquatic environments that are compromised because these measurements do not identify the biologically available dose. The biological endpoints that are measured are important because they relate to known impairments; however, they are not specific to the contaminants and often focus on only a few known endpoints. These studies can be enhanced by looking more broadly at changes in gene expression, especially if the analysis focuses on biochemical pathways. The present study was designed to obtain additional information for well-characterized sites adjacent to sewage treatment plants in MN that are thought to be impacted by endocrine disruptors.

**Results:**

Here we examine five sites that have been previously characterized and examine changes in gene expression in fathead minnows (*Pimephales promelas*) that have been caged for 48 h in each of the aquatic environments. We find that the gene expression changes are characteristic and unique at each of the five sites. Also, fish exposed to two of the sites, 7 and 12, present a more aggressive behavior compared to control fish.

**Conclusion:**

Our results show that a short-term exposure to sewage treatment plant effluents was able to induce a site-specific gene expression pattern in the fathead minnow gonad and liver. The short-term exposure was also enough to affect fish sexual behavior. Our results also show that microarray analysis can be very useful at determining potential exposure to chemicals, and could be used routinely as a tool for environmental monitoring.

## Background

Effluents from sewage treatment plants are a big source of endocrine-disrupting compounds (EDCs) into the aquatic environment. Exposure of aquatic organisms to EDCs has been linked to adverse physiological effects such as reduced fertility [1], intersex fish [2], sex reversal [3], and immunotoxicity or altered metabolism [4]. Effluents are very complex and variable mixtures of many different compounds [5,6], as a result their adverse effects on wildlife are extremely difficult to predict.

Lee et al. [5,7] analyzed the effects and the presence of EDCs in several streams in Minnesota. Potential sources of EDCs in Minnesota are treated sewage (domestic and industrial) and runoff from agricultural or forested land.

In the Lee study [7], female and male carp were collected at each site and were analyzed for fish health and a series of biomarkers of endocrine disruption. These included measurements of gonadosomatic index (GSI), plasma hormone and vitellogenin (Vtg) levels, and gonad histopathology. GSI is an indicator of reproductive status and chemical exposure and it is related to reproductive success. Gonad histopathology consisted of microscopic examination for the presence of abnormalities, such as ceroid/lipofuscin deposits in the males. Cellular level abnormalities are often seen prior to macroscopic abnormalities and are used as a warning of sublethal health effects and are correlated with increased susceptibility to disease [7]. In the present study only male fish were included.

These studies indicated the presence of EDCs by analyzing biological characteristics, such as hormone and vitellogenin levels, in the common carp. The studies identified sewage treatment plant effluents as a potential source of EDCs. Additionally, fish located at sites upstream of sewage treatment plant effluent draining agricultural and forested land showed indications of EDCs. The present study was designed to obtain additional information using genomics for some of these well-characterized sites in MN that are thought to be impacted by EDCs.

Microarray analysis has developed in recent years to the point where it can be used to gain mechanistic understanding about how fish health is impacted by contaminants. This method provides an unbiased open assessment of the health of fish exposed to polluted aquatic environments. A few studies have used microarrays to analyze the gene expression signature in fish exposed to polluted field sites [8,9]. If these efforts continue to efficiently move ahead, the use of microarrays to evaluate contaminants could become an extremely useful tool in ecological risk assessment.

A variety of anthropogenic chemicals such as industrial chemicals, surfactants and pesticides are known to have some estrogenic potency [2,10,11]. But more recently, among the primary estrogenic components of sewage discharge the human estrogens 17β-estradiol (E_2_) and estrone (E_1_), and the pharmaceutical estrogen 17α-ethinylestradiol (EE_2_), found in birth control pills, have been identified [12]. It is also evident that effluent run off from confined animal feeding organizations (CAFO) introduces high levels of anabolic androgens (17α-trenbolone, and 17β-trenbolone) [13], estrogens (E_2 _and zearalenone) and progestins (melengestrol acetate) [3]. Minnesota is impacted by effluents from sewage treatment plants that deal with domestic and industrial wastes and by run off from agricultural or forested land, where there is an abundance of CAFOs.

Here we examine five sites that have been previously characterized by Lee et al. [5,7] by examining changes in gene expression in male fathead minnows (*Pimephales promelas *– FHM) that were caged for 48 h in the different aquatic environments. We also examined whether 48 h exposure to water from the five sites of interest affected the ability of males to compete for nests and females. For many fish, including the FHM, acquisition of spawning territory is a competitive process in which more aggressive males acquire and maintain spawning territories (i.e., nest sites), whereas subordinate males often fail to reproduce. Past studies indicated that exposure to environmental estrogens can lead to reduced ability of males to acquire nests, whereas exposure to androgens can increase the performance of aggressive behaviors and enhance nest acquisition [14].

We hypothesized that fish exposed to water downstream from sewage treatment plants will have a different gene expression signature than those exposed to upstream waters, probably showing a more estrogenic signature, as waters downstream of sewage treatment plants have been shown to be estrogenic in some cases [15,16]. For a 48 h exposure, the gene expression signature will be a snapshot that depends on the contaminants present in the effluent during the time of exposure, rather than an average over time. We also hypothesized that the short exposure would be long enough to change fish sexual behavior.

## Results

### Selection of field sites

**Figure 1:**
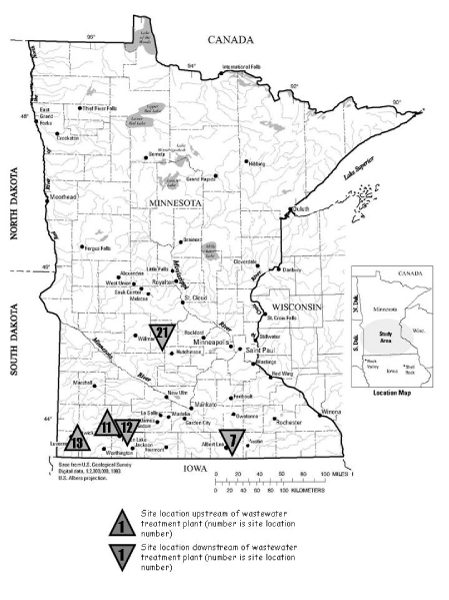
**Location of the sites**. Adapted from Lee *et al*. [5,7]

Five sites were selected for exposing FHM to possible EDCs in the natural environment (Figure 1 and Table 1). These sites were identified in Lee et al. [7] as showing evidence of EDCs using biological characteristics of common carp (*Cyprinus carpio*). The five sites were the following: Site 7 (43°36'02"; 93°17'30") is located on Shell Rock River near Albert, Lea, MN. Site 11 (43°51'54"; 95°18'47") is on the Des Moines River, upstream of Windom. Site 12 (43°51'27"; 95°06'28") is on the Des Moines River, downstream of Windom and down stream from a sewage treatment facility. Site 13 (43°43'04"; 96°09'49") is located on Rock River upstream of Luverne, MN. Although the high percentage of agricultural land and the number of animal feedlots in the drainage basin provides the potential for runoff of animal wastes to the streams, those characteristics do not guarantee that runoff into the stream has occurred or was present when the fish were exposed. A fifth site, Site 21 at Jewitt's Creek (45°08'42"; 94°31'00") downstream of Litchfield, MN, was identified in Lee et al. [5] by the presence of a number of sterols in the water quality analysis, although the effects in fish were not analyzed.

**Table 1:** Field sites characteristics

			Land use (% of drainage basin)						
**Site #**	**Site Name**	**Drainage area (km^2^)**	**Urban**	**Agicultural**	**Wetland**	**Forest**	**Water**	**Other**	**Feed Lots within 1 or 5 mi**

7	Shell Rock River near Albert Lea, MN	388	7.6	81.7	2.6	3.5	4.5	0.1	NA
11	Des Moines River upstream of Windom	1432	1.1	89.5	3.9	2.3	3.2	0	0, 3
12	Des Moines River down stream of Windom	2944	1.7	90.4	3.3	1.8	2.8	0	NA
13	Rock River upstream of Luvern, MN	792	1.3	94.9	2.2	1.5	0.1	0	2, 12
21	Jewitts Creek downstream of Litchfield, MN*	27	7.3	63.9	14.9	4.4	?	?	0, 1

Study sites and land basin characteristics from Lee et al. [7], Sites 3, 7, 11, 12, 13, and Lee et al. [5], Site 21. Feedlots are the number of "non-permitted" animal feedlots within approximately 1/4 mile from the stream or its tributaries within and 1 and 5 miles upstream of the exposure site.

Table was abstracted from Lee et al. [7]

* Data is from Lee et al. [7]

NA, data not available

### Microarray results

A 22,000 gene microarray specific for FHM [17] was used to analyze changes in gene expression patterns in liver and gonad of caged fish exposed to each of the effluents for 48 h. Although fish from site 11, a site located up-stream from a sewage treatment facility, were closest to laboratory controls (data not shown), gene expression patterns differed from the laboratory controls, probably due to differences in water quality and environment. This site is the closest to a reference site among the sites tested, yet it was not perfect. Fish at this site presented low GSI, but no Vtg induction in the males. Although we are aware that the site might have low concentrations of EDCs, it did have the lowest impact on fish, compared to the laboratory controls. Consequently, gene expression patterns at all of the field sites were compared to that of fish at site 11.

**Figure 2:**
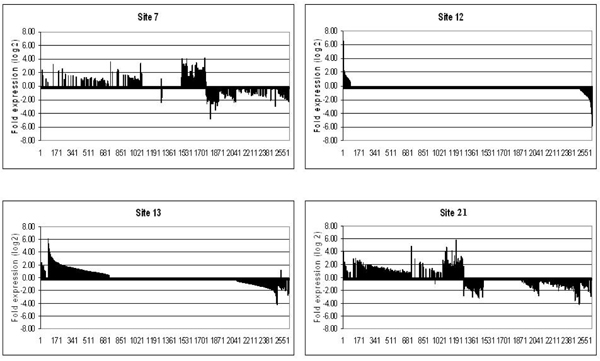
**Expression fingerprints for the liver at each of the sites**. Genes are expressed as fold change over expression at site #11 (similar to control). All genes were arranged from most highly expressed to most highly repressed for site#12 and the same order is kept for the other sites. Genes whose fold expression data was not significant at *p-*value < 0.05 were set to 0.

**Figure 3:**
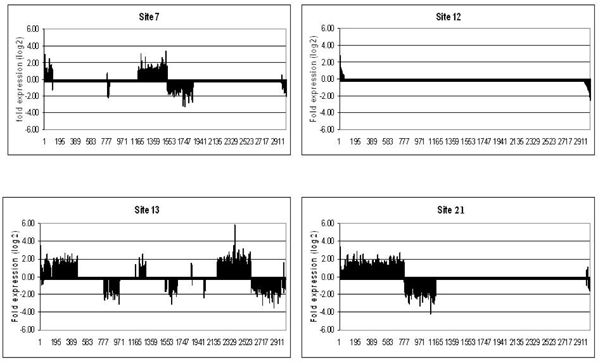
**Expression fingerprints for the gonad at each of the sites**. Genes are expressed as fold change over expression at site #11 (similar to control). All genes were arranged from most highly expressed to most highly repressed for site#12 and the same order is kept for the other sites. Genes whose fold expression data was not significant at *p-*value < 0.05 were set to 0.

To examine expression patterns across sites, we plotted the union of genes identified as significantly regulated across all sites (2624 total, *p*-value < 0.05) in relation to their fold-expression compared to Site 11 (Fig 2 and Fig 3). Each field site showed a unique gene expression pattern that was produced after only 48 h exposure of caged FHM. In the liver and the gonad, the number and identity of up- and down-regulated genes varied tremendously from site to site indicating the complexity of each of the effluents (Table 2).

**Table 2:** Gene expression changes

	LIVER			GONAD		
	up regulated	Down regulated	Total	up regulated	down regulated	Total

**Site 12**	80	133	213	48	64	112
**Site 7**	394	253	647	438	374	812
**Site 21**	434	431	865	738	388	1126
**Site 13**	650	463	1113	911	679	1590

Number of genes significantly altered compared to site 11

**Figure 4:**
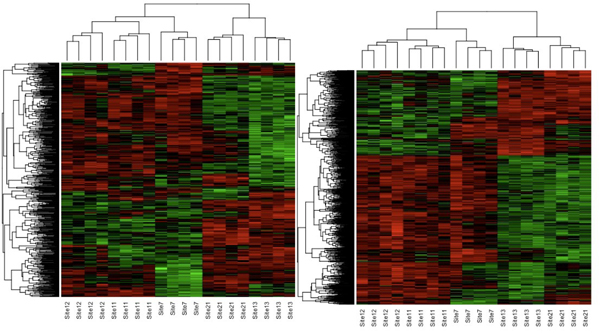
**Hierarchical clustering**. Two-way hierarchical clustering for genes differentially expressed following 48 h exposure at each of the sites. Expression data was analyzed by ANOVA and then z-transformed. Genes used in the cluster were significant at P < 0.05. Represented are genes that are up-regulated (red) or down regulated (green) by the treatments compared to controls. (A) Cluster for genes changed in the liver and (B) cluster for genes changed in the gonad.

Hierarchical clustering of all samples using the union of all differentially expressed genes was conducted to directly compare all the field samples (Fig 4A and 4B). It is clear that expression patterns grouped the fish together by location of exposure. Interestingly, the clustering patterns are very similar for both liver and gonad tissues. Sites 11 and 12, up- and down-stream respectively on the same river, had patterns of gene expression that were more clearly aligned with each other. Sites 7 and 12, both downstream locations with aggressive behavior, clustered together. Sites 21 and 13 clustered together, both sites with little or no impact on behavior after four days of testing despite being located on different rivers.

### Functional analysis

While it is interesting to identify individual genes regulated by each of the effluents, most biological processes occur through functional pathways. To assess this, we identified human homologs where possible and then used this information to find enriched Gene Ontology (GO) groups at each field site. We were able to identify human homologs for about 50% of the differentially expressed genes.

We used the functional clustering implemented in the web-based application DAVID (http://david.abcc.ncifcrf.gov, [18,19]) to determine significant changes in processes enriched in the differentially regulated gene sets in order to look at a higher order of complexity and determine which biological processes were altered because of the exposures (see additional files 1 to 4). The results show that exposure of fish to Site 7 waters alters processes involved in RNA splicing, metabolism, protein transport or protein catabolic process in both tissues, and also apoptosis in the liver. For site 12 some of the most enriched GO groups are sterol, cholesterol, and steroid biosynthetic process in the liver; and catabolism and signal transduction in the gonad. Another enriched GO group for the liver of site 12 was immune response, as previously described for the gonad of these fish [9]. Some of the main groups enriched for site 13 were metabolism, protein transport, or modification in the gonad; metabolism, mRNA processing or protein transport in the liver. For site 21, the main groups affected were protein transport, or metabolism in the gonad; and phosphorylation or steroid receptor signaling in the liver.

### Behavioral effects

**Figure 5:**
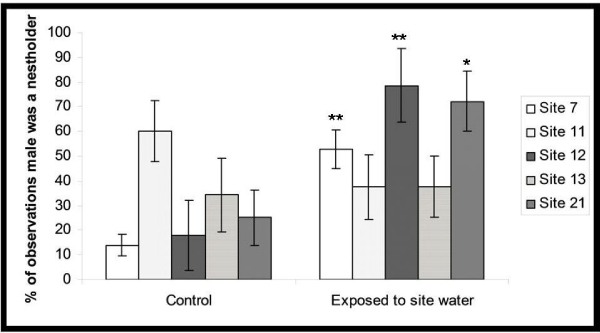
**Results of behavioral observations**. Asterices (*) indicate significant differences (t-test with Welch correction, P < 0.05) in the ability of males to occupy and defend nest over the period of the whole 4 (**) or just 2 (*) days of the test following a 48 h exposure period to site water.

The behavioral test found no effects on behavior in fish exposed at site 11, which lends support to the gene expression analyses that fish from the site were the least affected. Fish from site 13 also presented unchanged behavior. The fish from site 21 showed initial increases in aggressive behavior, but were not able to maintain possession of the nests; by the third day of behavioral experiments there were no significant differences in nest-holding ability between exposed and control males. Fish exposed to Sites 7 and 12 consistently dominated control fish and actively defended the majority of the nests over the course of the whole experiment. Figure 5 shows the results of behavioral observations.

## Discussion

These studies were designed to re-examine specific locations in rivers in Minnesota that had previously been studied by Lee et al. [5,7]. The Lee study design chose paired sites, targeting locations up stream and down stream of waste water treatment plants (WWTP) in Minnesota. The expectation was that up stream sites would be influenced more by agricultural run off and the down stream sites by effluent discharge and urban runoff. Locations on rivers were chosen that had a barrier (e. g. dam) that would prevent fish from swimming up stream. Originally the Lee et al. [7] study used 22 sites. Of these we picked 4 sites, those that seemed to have the most impact on fish reproduction. We picked an additional fifth site at Jewitt's Creek [5], labeled as site 21 located downstream of a wastewater treatment plant outfall. Of the original paired sites, we only have one on the Des Moines River near Windom; with site 11 (upstream) and site 12 (downstream) from the WWTP. The other sites selected were not paired. Effluents and consequently the waterways into which they disgorge are known to be very variable [5,6], therefore we expected that effects on fish might be different over time, and will depend on many factors, such as duration of exposure, season, or weather, among others.

### Gene expression and functional analysis

The expression fingerprints for fish exposed to five different field locations showed unique patterns for fish within each location that differed among the five regions picked (sites 7, 11, 12, 13 and 21) for both the liver and the gonad. Fish exposed at the same site showed similar gene expression profiles. Sites 11 and 12 were closely related consistent with their locations on the same river, up-stream and down-stream respectively, from a sewage treatment facility.

Site 11 male carp had the fewest biological anomalies; presenting only low GSI [7]. FHM exposed to this effluent for 48 h clustered most closely with non-exposed laboratory controls, suggesting that the water at this upstream location was not heavily contaminated [9]. At site 12, downstream from the WWTP site, male carp in the Lee study [7] also presented a low GSI in addition to lower plasma levels of 11-keto testosterone (11-KT) with an average of 400 pg/mL in fish from site 12 compared to an average of 600 pg/mL in fish from site 11. Carp also showed evidence of high ceroid/lipofuscin staining in their gonads, suggestive of susceptibility to disease. Some of the most enriched GO groups in FHM at site 12 were sterol and cholesterol biosynthetic process and steroid metabolism, all of which might help explain the decrease in 11-KT levels in relation to site 11, in endogenous carp. FHM placed in cages for 48 h at this location showed changes in gene expression that indicated that apoptosis or immune response were among the most affected GO groups in the liver (Additional file 2).

Site 7 is located on the Shell Rock River down stream from the WWTP near Albert Lea, MN. Male carp at this location [7] were shown to have plasma Vtg present. The levels of 11-KT in the carp were also lower than site 11 (400 pg/mL), and E_2 _levels were slightly higher (250 pg/mL compared to 150 pg/mL). In the present study, responses to site 7 appeared very complex, since we had from 650–800 genes altered by the treatment, but distinguishable from other sites by its molecular fingerprints. We did not find Vtg mRNA up-regulated after 48 h. We expect this site would be highly variable as well because it not only receives effluent from the sewage treatment facility but also because it gets a lot of agricultural runoff.

Males normally have a balance between estrogen and androgen hormones. Both types of hormones play a very important role in homeostasis. In site 7, we found several affected genes (prohibitin, prohibitin 2, and estrogen receptor-related gamma) that could be decreasing the effects of estrogenic compounds, thereby tipping the hormonal balance towards more androgenic pathways and resulting in more aggressive behavior. Prohibitin and prohibitin 2 were up-regulated both in the gonad and liver of fish exposed to site 7 waters. Prohibitin, a potential tumor suppressor, has been shown to function as a potent transcriptional corepressor for estrogen receptor alpha (ERα) [20]. The repression of ERα could help explain the aggressive behavior found in these fish. Estrogen receptor-related gamma (ERRγ) is down-regulated in the gonad of site 7. ERRγ is a member of the orphan nuclear receptor family. It does not bind to endogenous estrogens but it has been shown to bind to endocrine disruptors like bisphenol A and is deactivated by the ER antagonist [21] suggesting the presence anti-estrogenic compounds that might affect behavior. All these changes could help explain why Vtg mRNA was unchanged after a 48 h exposure.

Site 13, located upstream from Luverne, MN, has a considerable number of animal feeding operations within 5 miles of the site. Male carp at this location presented with high plasma E_2 _and Vtg levels and low plasma 11-KT levels, suggesting impairment in steroidogenesis and potential exposure to estrogenic contaminants [7]. They also showed evidence of high ceroid/lipofuscin staining in their gonads. Caged FHM in our study failed to show Vtg increases but did have the highest number of differentially expressed genes (1100–1600 genes). One of the genes up-regulated in the gonad and the liver of fish exposed to site 13 is progesterone receptor, which requires estrogen to be induced [22], suggesting that estrogenic compounds could be coming from the nearby CAFO facilities. The abnormal hormone levels found in fish exposed to this site could be related to the up-regulation of StAR (steroidogenic acute regulatory protein), a protein in charge of cholesterol transport into the mitochondria, a key step in steroidogenesis. Another up-regulated gene in the gonads of site 13 was the retinoic acid-related orphan receptor alpha (RORα), an orphan member of the subfamily 1 of nuclear hormone receptors. Cholesterol or cholesterol-derivatives are suspected to be its natural ligands of RORα, suggesting that RORα plays a very important role in the regulation of cholesterol homeostasis in humans [23]. There is also evidence that RORα participates in the xenobiotic regulatory network [24]. RORα up-regulation would also be consistent with the up-regulation of StAR, as it has been shown to be an important regulator of that protein in the largemouth bass [25].

Finally site 21, located on Jewitt's Creek near Litchfield, was chosen based on a separate study by Lee et al. [5]. It was chosen because it was within one mile of the discharge source and was the site that presented more EDCs and organic wastewater compounds from all the sites examined [5]. Exposure of FHM to this site altered 900–1100 genes. The most highly up-regulated protein in the liver was retinol binding protein (RBP). RBP up-regulation has been related to exposure to estrogenic compounds in *Xenopus laevis *[26,27]. Signal transducer and activator of transcription 1 (STAT1) is up-regulated in the gonad. STAT1 is a critical transcription factor involved in the JAK-STAT signalling pathway which is central for innate immunity [28] and apoptosis [29] among other functions. STAT1 is activated via the retinoic receptor signalling pathways [30] and it has been shown to be up-regulated by exposure to EE_2 _in the gonad of FHM [31]. This could help support the idea that exposure to estrogenic compounds decreased the initial aggressive behavior in fish from this site. The very dynamic nature of the effluent could have initially exposed fish to androgenic compounds immediately followed the day after by estrogenic compounds. The steroid hormone receptor signaling pathway was one of the most affected GO groups in the liver of these fish, which would also support that statement.

### Behavioral effects

The fish from Sites 7 and 12 were more successful at acquiring nests than control males, while fish from Site 13 showed no significant changes in behavior. The observed increases in the ability of males from sites 7 and 12 to occupy and defend nests are consistent with findings of others who found that waterborne androgens increased aggressive behavior and nest acquisition [14]. The fish from site 21 showed initial increases in aggressive behavior, but were not able to maintain possession of the nests; by the third day of behavioral experiments there were no significant differences in nest-holding ability between exposed and control males.

#### Unchanged or intermediate behavior

Sites 13 and 21 presented up-regulation of several thyroid hormone associated proteins (TRAP), both in the gonad (TRAP5 in both sites and TRAP230 in site 13) and the liver (TRAP3 in both sites and TRAP4 in site 21). The TRAP complex is a coactivator complex that interacts and modulates the activity of thyroid hormone receptors [32,33], suggesting the presence at both these sites of compounds that interact with the thyroid receptor. Disturbances in the thyroid system are associated with impaired reproduction in fish [34]. Flame retardants such as PBDE 47 (2,3,4,4-tetrabromodiphenyl ether) are known to affect thyroid hormone levels [35]. PBDE 47 has also been shown to decrease the number of mature spermatozoa in the FHM as well as the overall male reproductive fitness [35,36]. Although we do not have the flame retardant levels for site 13, nor the specific concentration of PBDE 47 in site 21, we do know that flame retardants were quite abundant in site 21 [5]. That might be related to the time-dependent decrease of aggressive behavior in fish from site 21 and the lack of aggressive behavior in fish from site 13.

CYP20A1 is up-regulated in these sites although that is not the case of some of the other CYPs involved in the metabolism of xenobiotics compounds, like CYPA1. The lack of CYP1A induction could also be related to the presence of flame retardants, as some have been shown to be antagonists that can reduce the induction of CYP1A by a more potent agonist [37,38].

Ornithine decarboxylase antizyme 2, an ornithine decarboxylase (ODC) inhibitor [39], was up-regulated in all sites. ODC has been identified as a sensitive marker of the action of androgens and antiandrogens in the testis [40]. Androgens are essential for the maintenance of ODC activity and administration of androgens increases synthesis of ODC mRNA [41]. ODC is involved in the biosynthesis of polyamines many of which (such as putrescine, spermidine, spermine) play an important role in reproduction [42]. Our finding that ODC antizyme 2 was up-regulated suggests that androgens were present in all sites and raise concern that the reproduction of endogenous fish at these locations may be impacted.

#### Aggressive behavior

In order to elucidate the causes of the increased nest acquisition rates (these are mediated by aggressive behavior), we compared the differentially expressed genes from sites 7 and 12. We expected these changes to be related to androgen exposure, as circulating androgens have been related to aggressive behavior in fish [43,44]. Thirty-one genes were common between site 7 and 12. Some of these genes were calbindin 2, Rho Family GTPase 3, NADH dehydrogenase subunit 2, caveolin 1 (CAV1), all up-regulated; and calmodulin, synaptogyrin 2, silencing mediator of retinoic acid and thyroid hormone receptor (SMRT), and hepatocyte nuclear factor 4 alpha (HNF4A), all down-regulated.

CAV1, a membrane protein up-regulated at sites 7 and 12, is one of the few proteins known to bind cholesterol, the precursor of steroid hormones, tightly and specifically. It has a very important role in cholesterol homeostasis. Caveolins have also been linked to reverse cholesterol transport, where excess free cholesterol is released into the plasma [45]. CAV1 has been previously found to respond to androgenic compounds [17], confirming the importance of this gene in relation to androgen exposure. RORα is down-regulated in sites 7 and 12. As mentioned earlier, this receptor is an important regulator for StAR and therefore, very important for steroidogenesis.

## Conclusion

Our results show that even a short exposure (48 h) to streams adjacent to sewage treatment plants was able to induce a site-specific gene expression pattern in the fathead minnow gonads and livers. The short-term exposure was also enough to affect the fish sexual behavior at two of the sites. These findings suggest that gene array analysis can complement chemical analysis as a monitoring tool. These findings also suggest that microarray analysis is relatively robust especially when used in conjunction with other more established methods to define contaminated aquatic environments for risk assessment and environmental monitoring.

## Methods

### Fish exposures

To conduct the field exposures at each field site, FHM were transported from the laboratory to the field site in aerated, insulated tanks. At each site, 25 males and 25 females were placed in separate wire mesh minnow traps with the entrance funnel plugged. The traps were anchored to the bottom in the stream current with the top of the traps submerged. Fish were removed from the traps 48 h later and transported back to the laboratory in aerated, insulated tanks containing the stream water. Immediately upon arrival at the laboratory, four males and four females were sacrificed as described for the laboratory exposures. Liver and gonads were removed and stored in liquid nitrogen until processed for arrays.

All procedures involving live fish were reviewed and approved by the University of Minnesota Institutional Animal Care and Use Committee (IACUC).

#### Preparation of total RNA

Total RNA was isolated from gonadal tissue with the RNA Stat-60 reagent (Tel-test, Friendswood, TX) as described previously [46]. RNA pellets were resuspended in 50 to 150 μl RNA Secure (Ambion, Austin, TX) to inactivate RNases following the manufacturer's protocol. A total of 10 μg of RNA was treated with DNase to avoid contaminating DNA using DNA-*free *(Ambion, Austin, TX) following the manufacturer's protocol. The quality of total RNA was assessed with the Agilent 2100 BioAnalyzer (Agilent, Palo Alto, CA) and the quantity was determined on a NanoDrop spectrophotometer (NanoDrop Technologies, Wilmington, DE). RNA was stored at -80°C until further use.

### Microarrays

Fathead minnow 22,000 gene arrays were designed by EcoArray (Alachua, FL) and were purchased from Agilent. Array hybridizations were performed using a reference design, where each sample was compared to a reference sample. The reference sample consisted of equal amounts of RNA from control female and male tissues (liver, brain and gonad). Four replicates, each consisting of a different individual, were analyzed for each of the treatment sites (sites 7, 11, 12, 13 and 21). cDNA synthesis, cRNA labeling, amplification and hybridization were performed following the manufacturer's kits and protocols (Agilent Low RNA Input Fluorescent Linear Amplification Kit and Agilent 60-mer oligo microarray processing protocol; Agilent, Palo Alto, CA). Gonad and liver samples from the fish at the sites were labeled with Cy5 while the reference sample was labeled with Cy3. Consistent with the Minimum Information about a Microarray Experiment (MIAME) standards [47], text versions of the Agilent raw data from this study have been deposited at the Gene Expression Omnibus website (GEO: http://www.ncbi.nlm.nih.gov/geo/; Accession series record number GSE16645).

### Bioinformatics

Microarray image processing and data pre-processing were performed using Agilent's Feature Extraction software v 9.5 (Agilent, 2007). The intensity of each spot was summarized by the median pixel intensity. A log_2 _transformed signal ratio between the experimental channel and the reference channel was calculated for each spot, followed by within-array lowess transformation and between array scale normalization on median intensities [48].

One-way ANOVA was performed on normalized log_2 _transformed signal ratios of each probe individually, followed by Tukey-HSD pair-wise comparisons to determine genes whose expression was significantly regulated between sites. Statistical significance was determined at a *p*-value of < 0.05 with an FDR threshold of 16%. FDR was calculated using Benjamini-Hochberg approach [49]. After testing for significance we also eliminated from consideration genes whose fold-expression changes were less than 1.5 fold.

### Behavioral assay

Individual fish were transferred into 10-L glass test aquaria for behavioral testing, each of which was supplied with clean well water (50 ml/min). Prior to placement in tanks, both site water-exposed and control males were acclimated to the temperature of the observation tank (25°C) over a 4-h period. For each one of the sites, we tested 7–10 behavioral replicates. Each one of these replicates consisted of a control male added along with the site water-exposed male to an aquaria containing one unexposed female and a nest. Male fish were selected so that their total lengths were within 3 mm of each other and one fish had a upper caudal fin clip, and the other a lower caudal fin clip (marking pattern was not treatment-specific).

The behavioral assay followed protocols described by Martinovic et al. [14]. Briefly, identification of nest-holders commenced after 24 h had elapsed from the time the fish were introduced into test aquaria. Each individual was observed each day between 10.00 h and 14.00 h for 5 min and those fish which spent the majority of their time in a nest while also exhibiting the nest-tending (nibble, rub nest etc.) or nest-defense behaviors (chase, bite, butt etc.) were categorized as 'nest-holders'. After 4 days the experiments were terminated. We calculated the percentage of times an individual was identified as a nest-holder (number of times each male was identified as a nest-holder was divided by total number of observations). These data were tested using Kolmogorov and Smirnov test and because none of the datasets violated the normality assumption, the significance of differences in nest holding between controls versus exposed males was evaluated with unpaired t-test with Welch correction. In addition to examining overall nest-holding ability (over 4 days) we also compared the number of control and exposed nest holders for each day using Fisher's exact tests. The behavioral data are reported as significantly different if *p *< 0.05.

## Competing interests

The authors declare that they have no competing interests.

## Authors' contributions

NGR: RNA extraction, microarrays, drafted the manuscript; IRA: conceived of the study, and participated in its design and coordination and helped draft the manuscript; DM: behavior assays, helped draft the manuscript; LL: bioinformatics analysis, helped drafted the manuscript; NDD: conceived of the study, and participated in its design and coordination and helped draft the manuscript. All authors read and approved the final manuscript.

## Supplementary Material

Additional file 1 functional clustering of enriched GO biological processes altered in site # 7. Click here for file

Additional file 2 functional clustering of enriched GO biological processes altered in site # 12. Click here for file

Additional file 3 functional clustering of enriched GO biological processes altered in site # 13. Click here for file

Additional file 4 functional clustering of enriched GO biological processes altered in site # 21. Click here for file
